# The model of norm-regulated responsibility for proenvironmental behavior in the context of littering prevention

**DOI:** 10.1038/s41598-024-60047-0

**Published:** 2024-04-23

**Authors:** Pengya Ai, Sonny Rosenthal

**Affiliations:** Wee Kim Wee School of Communication and Information, Nanyang Technological University, Singapore, 637718 Singapore

**Keywords:** Environmental social sciences, Psychology and behaviour

## Abstract

Previous research suggests that descriptive norms positively influence proenvironmental behavior, including littering prevention. However, in some behavioral contexts, a weak descriptive norm may increase individuals’ feelings of responsibility by signaling a need for action. We examined this effect in the context of litter prevention by conducting structural equation modeling of survey data from 1400 Singapore residents. The results showed that descriptive norms negatively predicted ascription of responsibility and were negatively related to littering prevention behavior via ascription of responsibility and personal norms. It also showed that strong injunctive norms can reduce the inhibitory effect of descriptive norms on ascription of responsibility. These findings were consistent with several hypotheses constituting the model of norm-regulated responsibility, a novel explanatory framework offering new insights and a more nuanced and comprehensive understanding of social norms’ influence on proenvironmental behavior.

## Introduction

Littering stands as a stark reminder of the detrimental effects human actions can have on the environment^1^. Beyond its unsightly appearance within communities, littering has negative ecological, health, and economic consequences. It pollutes water bodies, disrupts ecosystems, poses a threat to wildlife, and potentially harms human health^2^. Despite knowing that littering is a problem, many people still litter, and individuals are the primary source of litter^3^. This is even true in Singapore, where the present study took place. It is a country known for cleanliness and large littering fines. Still, litter prevention remains a persistent challenge. In 2022, the National Environment Agency issued over 20,000 littering tickets, an increase over previous years^4^. In other countries, littering is also a problem. A 2020 survey reported that roadways and waterways in the United States are burdened with an alarming volume of litter, estimating nearly 50 billion individual pieces^5^. Thus, how to discourage littering has become an essential academic question and has practical economic, social, and environmental value. Research has explored factors and strategies influencing littering prevention behavior, among which social norms are a prominent feature^6^.

Social norms are rules, standards, expectations, and behaviors shared in a group^7,8^. Scholars often classify social norms as *descriptive norms* and *injunctive norms*. Descriptive norms refer to the observed or perceived behavior of group members^9,10^. These kinds of norms influence behavior by providing social information, which individuals can use to surmise the correct thing to do^11^. In contrast, injunctive norms refer to the anticipated approval or disapproval from others for engaging in a behavior^9,10^. Their effects hinge on people’s motivation to be accepted by others and meet social expectations^11^. Perhaps a simple way to understand the difference between the two kinds of norms is that descriptive norms indicate the “is” aspect of social norms, while injunctive norms suggest the “ought” aspect of them^9,10^. Therefore, people conform to descriptive norms for accurate decision-making and comply with injunctive norms for social approval^12^.

Studies have shown that stronger social norms result in more proenvironmental behavior (i.e., behaviors that aim to minimize people’s negative impacts on the natural and built environments, such as littering prevention)^13,14^ or at least a stronger behavioral intention^15–17^. In general, this should be the case with injunctive norms, which create tacit social boundaries of acceptable behavior. However, it is possible that strong descriptive norms may reduce behavioral motivations in some contexts. This is because the belief that other people engage in a behavior may suggest that enough is already being done to address a problem. Such an argument is reasonable in the context of proenvironmental behaviors, which often aim to resolve collective problems and require collective action^18,19^, and it is consistent with a meta-analysis showing that descriptive norms have a weaker effect than injunctive norms on behavior^20^. When a descriptive norm provides information that collective action may already be taking place, individuals may have less of a personal ascription of responsibility to act.

Ascription of responsibility is a key component of the norm activation model (NAM) and a direct predictor of personal norms—or feelings of moral obligation—to engage in helping behaviors^21^. Early research using that model was interested in the role of responsibility denial^22,23^, which is somewhat the inverse of ascription of responsibility, and may arise when individuals feel they did not contribute to a problem or that the needed course of action to fix a problem is out of their control^21^. As we hinted earlier and will argue later, responsibility denial may also result from a strong descriptive norm. To test that argument, we propose a serial mediation model, in which injunctive and descriptive norms predict proenvironmental behavior indirectly through ascription of responsibility and personal norms.

The potential negative path between descriptive norms and behavior creates what is in statistical terms an inconsistent mediation model, also called a suppression effect, which means that the signs of the direct and indirect relationships between an independent variable and a dependent variable are opposite^24^. On a conceptual level, this inconsistent mediation model can augment scholarly understanding of normative influence in certain behavioral contexts. Although incorporating social norms into the NAM is not new, little prior research examined the relationship between social norms and ascription of responsibility, and none has proposed inconsistent mediation effects on the ascription of responsibility (For the interest of some readers, Appendix 1 summarizes research about the role of social norms and ascription of responsibility in the context of littering). Moreover, we test the argument that the relationship between descriptive norms and the ascription of responsibility depends on the level of injunctive norms. Investigating the juxtaposition of descriptive norms and injunctive norms, rather than examining their effects separately, offers a unique way to study the impact of descriptive norms on proenvironmental behavior. This approach also helps to isolate the influence of injunctive norms. Furthermore, exploring the interaction of injunctive and descriptive norms is important because, in many contexts, they can work together to influence people’s behaviors. Thus, the present study can contribute a novel perspective to explaining proenvironmental behaviors, such as litter prevention.

## Literature review

### The norm activation model

The NAM uses the concept of personal norms to explain why people engage in prosocial behavior^21^. Personal norms reflect feelings of moral obligation to behave in a certain way. They are self-expectations about behavior and stand in contrast to the social expectations that social norms effect^21^. Whereas social norms (e.g., descriptive norms and injunctive norms) are external regulations that create more extrinsic motivations to act, personal norms are internal regulations, akin to intrinsic or more self-determined motivations^25^. There is growing evidence that personal norms are perhaps the most important predictor of proenvironmental behavior^26,27^, and there is value in understanding how individuals form personal norms.

**Figure 1:**
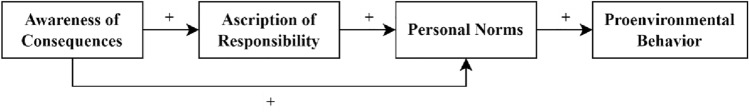
The norm activation model.

Schwartz^21^ identified four steps to activate personal norms. First, individuals must perceive that others are in need. Scholars generally refer to this as an awareness of consequences or problem awareness^28^. Second, individuals must be aware of actions that can help those in need. Third, individuals must recognize their own abilities to provide help. Fourth, individuals must have an ascription of *personal* responsibility, which draws on “a sense of connection or relatedness with the person in need”^21^ and results in individuals believing that resolving that need depends on their action. In the NAM, this concept is often called ascription of responsibly, which refers to the responsibility of individuals, including the self, in comparison with actors such as governments and corporations that may also share in the responsibility^29^. Compared with personal norms, which implicate a perceived duty to act, ascription of responsibility indicates a causal contribution to the consequences and is thus less morally rooted^30^. Through these four steps, individuals activate their personal norms, creating moral frameworks of action that motivate subsequent helping behaviors. Scholars have found support for the causal sequence of this model (see Fig. 1)^28,31^. Also, since both prosocial and proenvironmental behaviors have a moral basis^32^, the NAM appears widely in research in proenvironmental behavioral contexts, including recycling^33^, energy saving^34^, and electric vehicles adoption^35^.

#### Extensions of the norm activation model

In addition to using the NAM to explain proenvironmental behaviors, scholars have extended it to incorporate social norms, mainly following two approaches. According to the first approach, personal norms arise when individuals internalize social norms^21^. Through this process, the motivation to comply with social norms takes on a more self-directed character over time. Thus, rather than engaging in a behavior to avoid social exclusion, which is an extrinsic motivator, individuals may engage in behaviors out of a need for self-consistency or, when they fully internalize social norms, as an end in itself and an intrinsic motivator^36,37^. Consistent with the notion of norm-internalization, there is evidence that personal norms mediate the relationship between social norms and proenvironmental behavior^38–40^.

**Figure 2:**
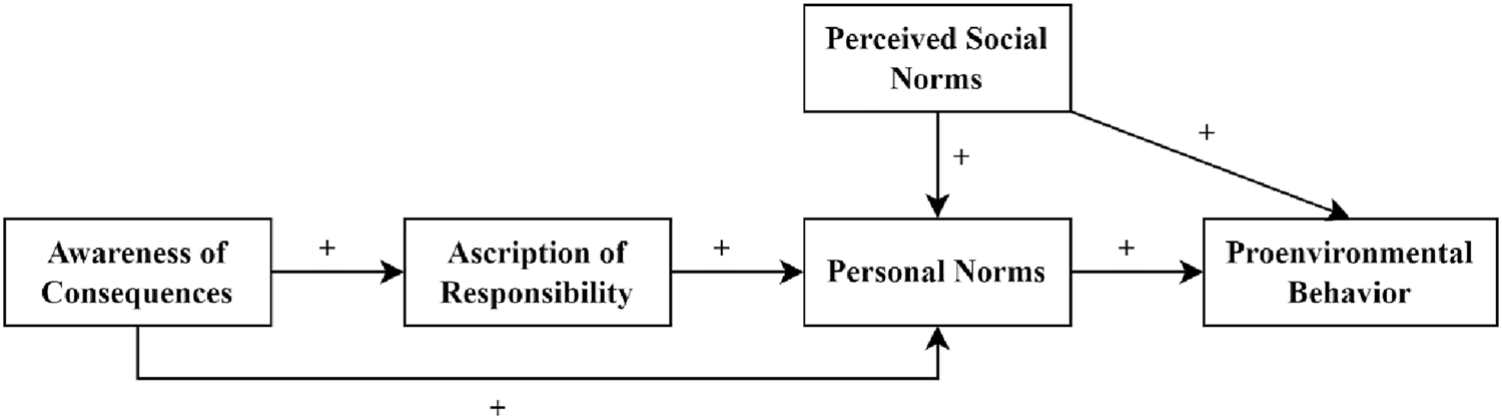
An extended norm activation model.

The second approach to incorporate social norms into the NAM is by combining the NAM with the theory of planned behavior (Ajzen, 1991). According to the theory of planned behavior, individuals form the intention to engage in a behavior when they hold positive attitudes toward it, perceive social norms encouraging it, and believe they have personal control over its performance. By combining the two models, the three predictors from the theory of planned behavior are parallel to, rather than in sequence with, personal norms. Some scholars have referred to this combination as an extended theory of planned behavior with personal norms as a fourth predictor of behavioral intention^41^. Although many studies have modeled social norms and personal norms as strictly parallel predictors e.g.,^29,42^, some also treated perceived social norms as a predictor of personal norms e.g.,^43,44^. That latter linkage is consistent with the norm-internalization argument^36,37^, which blurs the distinction between the two approaches and creates a more holistic, albeit less parsimonious, model of normative influence (see Fig. 2).

There is a third approach that does not augment or extend the NAM, but rather examines the interplay of social norms and personal norms. According to that approach, the effects of social norms depend on the level of personal norms. That idea draws partly on research which showed that subjective norms influenced behavior only when individuals had low personal involvement^45^. Testing such an effect of personal norms, at least two studies have shown that perceived social norms influenced proenvironmental behaviors only when personal norms were weak^46,47^. We acknowledge this important research, but also note that the model it presents is quite different from the norm-internalization argument central to the present work. The fact that personal norms may moderate the effect of social norms does not rule out norm-internalization.

### Ascription of responsibility vis-à-vis social norms

Despite the evidence linking social norms, personal norms, and proenvironmental behavior, prior theorization overlooks an important distinction between descriptive and injunctive norms related to the regulation of ascription of responsibility. We argue that descriptive norms may contribute to a denial of personal responsibility in the context of helping behaviors, including proenvironmental behavior, whereas injunctive norms create a moral imperative to act and will enhance a sense of personal responsibility.

Proenvironmental behaviors generally concern the management of public goods like clear air and water^18^. This means everyone is free to access the resources from the environment. This makes public goods management the responsibility of everyone, but also nobody in particular, which may create ambiguity over who should manage those goods^48^. Scholars have identified this phenomenon as one type of social dilemma^18^. Free access also means that individuals share the benefits of each other’s proenvironmental behaviors, which could result in a sort of bystander effect, where an individual may avoid helping out if they perceive that others are available to step in. As descriptive norms serve as behavioral information by definition, a strong descriptive norm of proenvironmental behavior indicates the belief that many other people have engaged in protecting the environment. That belief could trigger a bystander effect, reducing the ascription of responsibility over managing public goods.

There are at least two pathways to that effect. First, from the social dilemma perspective, when many people act or are at least perceived to act to sustainably manage a public good, it reduces the presumed necessity of any one individual acting. So, to an individual, strong descriptive norms reduce the sense of personal responsibility. Second, the belief that few people engage in proenvironmental behavior may signal the failure of collective action or the inability or unwillingness of others to contribute. That is, weak descriptive norms threaten the public good. That creates an urgency for individuals to assume responsibility^49,50^ and makes it more difficult for them to shift the responsibility to others^48^. Both pathways suggest a negative relationship between descriptive norms and ascription of responsibility, which we predict:

#### Hypothesis 1:

There is a negative relationship between descriptive norms about a littering prevention behavior and ascription of responsibility for that behavior.

Compared to the relationship between descriptive norms and ascription of responsibility, the linkage between injunctive norms and ascription of responsibility is straightforward. Because injunctive norms establish what *should* be done rather than describing what *is* done^8,51^, they can trigger feelings of personal responsibility and establish a moral basis of action. Believing there are social sanctions for violating norm-implied social expectations can further enhance a sense of personal responsibility^52^. This would suggest a positive relationship between injunctive norms and ascription of responsibility, which we predict:

#### Hypothesis 2:

There is a positive relationship between injunctive norms about a littering prevention behavior and ascription of responsibility for that behavior.

Moreover, we suggest descriptive and injunctive norms may have an interaction effect on ascription of responsibility. Scholars have argued that injunctive norms can amplify the positive effect of descriptive norms on behavior by making norm-noncompliance riskier in terms of social punishment^51,53^. Göckeritz, et al.^45^ provided evidence of that effect on self-reported energy conservation behavior. However, if strong descriptive norms result in weaker ascription of responsibility, which is consistent with our first hypothesis, then what is the role of injunctive norms? We think they have a similar role, but instead of amplifying a positive effect, they attenuate a negative effect of descriptive norms. In addition to making norm-noncompliance riskier, injunctive norms may minimize the diffusion of responsibility caused by strong descriptive norms. By the same argument, weak injunctive norms will support the diffusion of responsibility that strong descriptive norms may trigger because there would be less social expectation to act. Such an argument is partially supported by Habib et al.^50^, who found that the combination of low descriptive norms and high injunctive norms is more effective in influencing personal responsibility than simply low descriptive norms or high injunctive norms messages in the organ donation context. However, their study design did not allow them to demonstrate the interaction effect of descriptive and injunctive norms, which the current study proposes.

#### Hypothesis 3:

The stronger the injunctive norms, the less negative the relationship between descriptive norms and ascription of responsibility.

### Indirect effects on personal norms and littering prevention behavior

There is a well-established causal relationship between ascription of responsibility and personal norms^28^. Thus, our argument that descriptive norms are negatively related to ascription of responsibility may seem to imply a similarly negative relationship between descriptive norms and personal norms. Such an implication would contradict the norm internalization process we described earlier. However, it is possible that descriptive norms have a negative indirect relationship with personal norms via ascription of responsibility but a positive direct relationship. This is because ascription of responsibility involves a more superficial sense of who should act to address a need, which is in contrast with the deeper moral basis personal norms may create. Thus, whereas strong descriptive norms may indicate that others are already addressing a need and reduce the sense of urgency about it, they may also contribute to the belief that addressing the need is the moral course of action. Individuals in this situation might think, “I do not need to take action, but it would be good if I did.” These contradicting pathways between descriptive norms and personal norms suggest an inconsistent mediation (i.e., suppression) effect, which we predict:

#### Hypothesis 4:

There is a positive direct relationship between descriptive norms and personal norms and a negative indirect relationship between them via ascription of responsibility.

Furthermore, there ought to be a positive relationship between injunctive norms and personal norms, both directly via norm internalization and indirectly via ascription of responsibility. This establishes a standard mediation effect, which we predict:

#### Hypothesis 5:

There is a positive relationship between injunctive norms and personal norms, which ascription of responsibility mediates.

As research has established a strong linkage between personal norms and proenvironmental behavior^14,54,55^, the predicted mediation effects on personal norms ought to be transmitted to proenvironmental behavior, too. Such linkages would support a novel causal pathway from descriptive and injunctive norms to proenvironmental behavior via their opposing effects on ascription of responsibility. Thus, we predict:

#### Hypothesis 6:

There is a negative serial indirect relationship between descriptive norms and littering prevention behavior via ascription of responsibility and personal norms.

#### Hypothesis 7:

There is a positive serial indirect relationship between injunctive norms and littering prevention behavior via ascription of responsibility and personal norms.

Finally, we expect that the indirect effects of descriptive norms on personal norms and proenvironmental behavior via ascription of responsibility are moderated by injunctive norms. Such moderated mediation presumes that the interaction effect between descriptive norms and injunctive norms on ascription of responsibility, which we predicted earlier, transmits to personal norms and then to proenvironmental behavior.

#### Hypothesis 8:

The stronger the injunctive norms, the less negative the indirect relationship between descriptive norms and personal norms via ascription of responsibility.

#### Hypothesis 9:

The stronger the injunctive norms, the less negative the serial indirect relationship between descriptive norms and littering prevention behavior via ascription of responsibility and personal norms.

**Figure 3:**
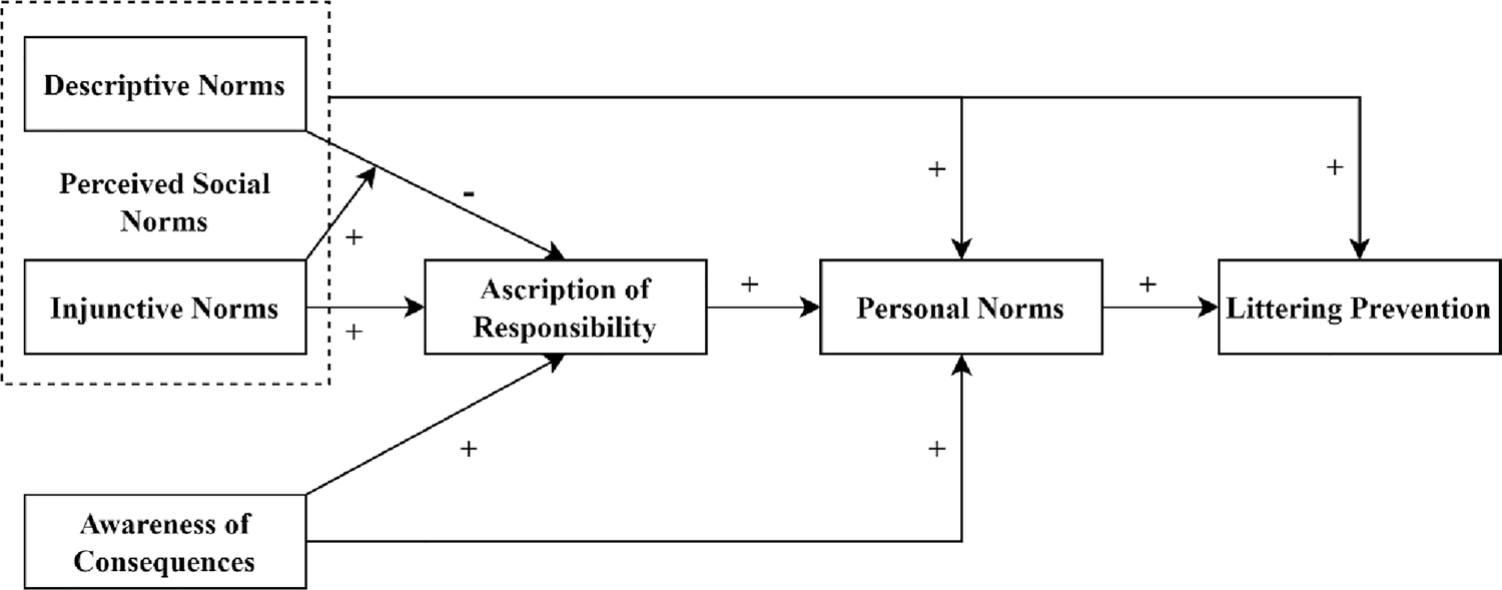
The model of norm-regulated responsibility.

These predictions suggest a novel way of incorporating social norms into the NAM. This model, the model of norm-regulated responsibility, has mostly the same elements as the extended NAM, but it focuses on explaining ascription of responsibility and thus includes novel pathways. Unlike the extended norm activation model, where social norms are positively related to personal norms, the model of norm-regulated responsibility highlights additive and synergistic effects of potentially incongruent descriptive and injunctive norms (see Fig. 3).

## Method

### Data source and context

We used an existing representative survey of Singapore residents to test this model. The main purpose of that survey was to text an extended NAM^56^, but it included several additional measures that could be used in secondary analyses. Those additional measures included descriptive and injunctive norms, which have not been included in any published analyses of those survey data. That dataset was appropriate for testing the current model because the behavioral measures concerned littering prevention, specifically the actions individuals take to avoid contributing to litter by picking up after themselves. This type of behavior concerns a social dilemma about a public good. Although Singapore generally has little visible litter, much of that cleanness is due to regular litter collection by the government, which consumes public resources^56^.

Data collection involved door-to-door survey of residents in public housing blocks, where at the time of data collection roughly 80% of Singapore residents lived^57^. It employed multistage cluster sampling, drawing a random sample of public housing blocks within a random sample of neighborhood within five regions of Singapore. The number of housing blocks selected were proportional to the population of each region and neighborhood. All households were sampled within each selected block and the “last birthday” method was used to sample the adult resident who most recently had their birthday^58^. These procedures adhered to the guidelines and regulations of the Institutional Review Board at Nanyang Technological University, Singapore (approval number: IRB-2018-02-030). All participants provided informed consent for both participation in the study and the responsible sharing of their data for academic and scientific dissemination.

The survey was completed by 1,400 Singaporean adults over two weeks in July and August 2018. Among those respondents, most of those participants (n = 1312) received paper surveys, which they could fill out using a writing implement. Some participants (n = 79) had difficulty reading and requested the research assistant read them the questionnaire. A few participants (n = 9) requested an online version, which they completed on Qualtrics. The research assistants knocked on the doors of 8,180 residences (a response rate of 17%) and someone answered the door at 3,812 of them (a completion rate of 37%). The sample consisted of 86.6% Singapore citizens, 6.8% permanent residents, and 4.6% foreigners. There were more females (52.6%) than males (46.0%). The majority of the respondents were Chinese (71.4%), followed by Malay (12.9%), Indian (10.6%), and other ethnic groups (3.7%). The median age group was 36 to 40 years old. The median monthly income bracket was 5,000 to 5,999 Singapore dollars. These demographics were similar to official census figures^59^.

### Measurements

The survey adapted prior measures of descriptive norms^60,61^, injunctive norms^60,61^, ascription of responsibility^62^, and personal norms^63^, and used five-point Likert scales ranging from 1 (*strongly disagree*) to 5 (*strongly agree*). The measurement of littering prevention behavior employed hypothetical scenarios in which the respondents accidentally littered. Then, they rated the likelihood of them picking up the litter on a scale ranging from 1 (*definitely no*) to 5 (*definitely yes*). The purpose of using hypothetical scenarios was to reduce the tendency to respond in a socially desirable way, given that the questions were about a positive behavior^64^. The wording and descriptive statistics of each item appear in Appendix 2. We used awareness of consequences^65^ as a control variable predicting ascription of responsibility and personal norm as informed by the NAM.

To address potential common method bias, we calculated the variance inflation factor (VIF) for each latent construct included as exogenous variable or mediator. None of the resulting values (see Appendix 2) exceeded 3.3, which according to Kock^66^ is the threshold indicating "pathological collinearity" suggestive of common method bias. Although we cannot rule out common method bias with this test, it suggests that the common method bias was not problematic in this study.

We estimated our model using structural equation modeling (SEM). We opted for SEM over other data analysis methods for at least three reasons. First, SEM involves the analysis of latent variables, which reflect common variance among the measurement items and can minimize measurement bias^67^. Second, it allows for the estimation of multiple parallel and serial regression paths, including mediation and moderation, which is necessary for us to test our hypotheses^67^. There are certainly other approaches that also allow this, but they lack the benefit of using latent variables. Third, SEM is widely used in the analysis of environmental psychology models^14,29,68,69^.

## Results

According to the criteria by Hu and Bentler^70^, the measurement model had a good fit, χ^2^(90) = 234.54, CFI = 0.98, RMSEA = 0.03, 90% CI [0.03, 0.04], SRMR = 0.02. There were no factor cross-loadings or correlated item residuals. The average variance extracted (AVE) was above 0.5 for all constructs, and the square roots of the AVE of each construct was larger than its correlation with any other construct, suggesting acceptable convergent and discriminant validity^71^. See Appendix 2 for the factor loadings, average variance extracted, and composite reliability, and Appendix 3 for the correlations among the model variables.

### Main effects

**Figure 4:**
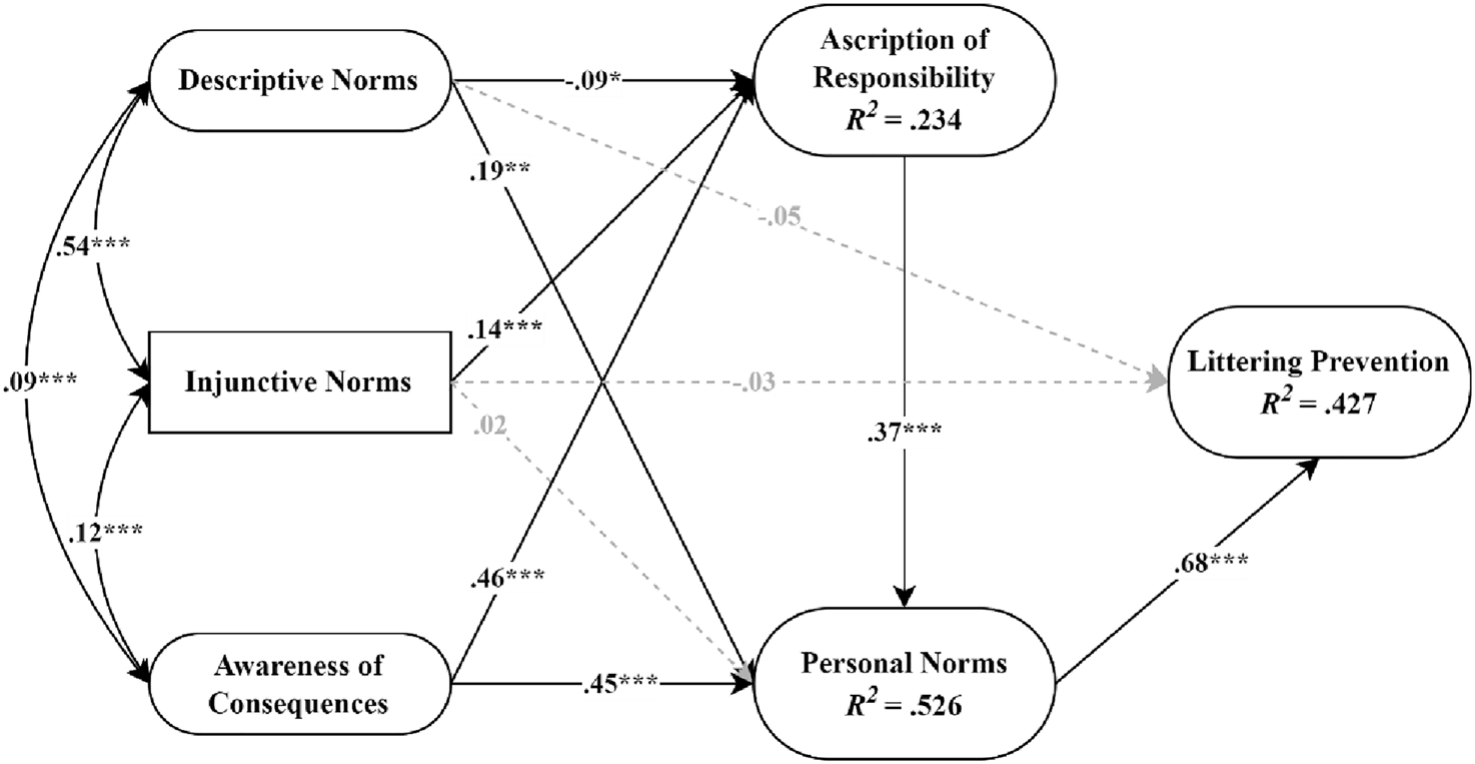
Standardized path estimates of the baseline model. *p < .05, **p < .01, ***p < .001. Ovals represent latent variables and rectangles represent observed variables.

The structural model included the hypothesized paths. Descriptive norms, injunctive norms, and personal norms as predictors of littering prevention. This model had good fit, χ^2^(92) = 236.46, CFI = 0.98, RMSEA = 0.03, 90% CI [0.03, 0.04], SRMR = 0.02. Figure 4 shows the standardized path estimates of the structural model (see Appendix 4 for the standardized coefficients, *p*-values, and bootstrapped 95% confidence intervals of the direct and indirect effects).

Consistent with hypotheses 1 and 2, descriptive norms were negatively related (*β* = -0.09, *p* = 0.02) and injunctive norms were positively related (*β* = 0.14, *p* < 0.001) to ascription of responsibility.

### Interaction effect

We estimated the two-way interaction of descriptive norms and injunctive norms. The latent moderated structural equation method in Mplus does not include fit indices such as CFI, RMSEA, and SRMR, so we could not directly assess the fit of the interaction model. Therefore, we used the two-step method recommended by Maslowsky, et al.^72^ to indirectly evaluate the model fit after adding the interaction term. This method is based on the log-likelihood ratio test. The index is denoted as *D*.

$$D\, = \, - {2}\left[ {\left( {{\text{log}} - {\text{likelihood for Model }}0} \right){-}\left( {{\text{log}} - {\text{likelihood for Model 1}}} \right)} \right].$$

Model 0 is the model without the interaction term, while Model 1 is the model with the interaction term. The value of *D* approximately follows a *χ*^2^ distribution and the degrees of freedom is the difference in the number of free parameters between the two models, which in this case is 1. A significant value of *D* would suggest that Model 1 fits well because the additional degree of freedom to achieve Model 0 results in a loss of fit. If the log-likelihood ratio test is not significant, it implies that Model 0 does not have worse fit than Model 1. In that situation, we cannot draw conclusions about the relative fit of the models, but it does not rule out Model 1 as well fitting.

**Figure 5:**
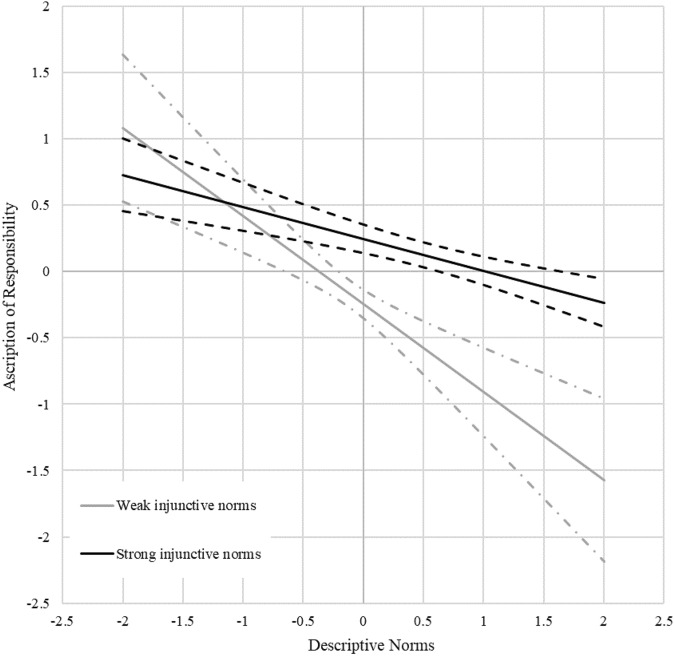
Pick-a-point plot of the interaction effect. Both axes show standard deviations from mean values. The solid lines show the conditional main effects of descriptive norm on ascription of responsibility at low and high values (*M* ± 2*SD*) of injunctive norm. The dashed lines show the 95% confidence intervals of conditional main effects.

**Figure 6:**
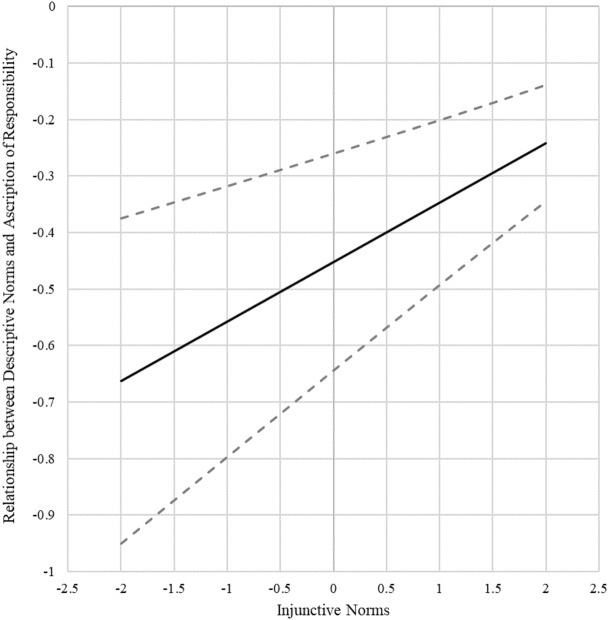
Johnson-Neyman plot of the interaction effects. The vertical axis shows the standardized relationship between descriptive norms and ascription of responsibility. The horizontal axis shows standard deviations from the mean on injunctive norms. The solid line shows the point estimates of the relationship between descriptive norms and ascription of responsibility for each increment of injunctive norms. The dashed lines show the 95% confidence interval of the point estimates.

Results showed a positive interaction effect (*β* = 0.10, *p* < 0.001), but the log likelihood ratio test was not significant (see Appendix 5). We plotted the interaction effect using the pick-a-point (see Fig. 5) and Johnson-Neyman (see Fig. 6) methods. Both figures show that the stronger the injunctive norms, the less negative the relationship between descriptive norms and ascription of responsibility. This pattern supports hypothesis 3.

### Indirect effects

Consistent with hypothesis 4, ascription of responsibility mediated the negative relationship between descriptive norms and personal norms (*β* = − 0.03, *p* = 0.02, 95% CI [− 0.06, -0.004]), while the direct relationship between descriptive norms and personal norms was positive (*β* = 0.12,* p* = 0.01, 95% CI [0.03, 0.20]). Similarly, the indirect relationship between descriptive norms and littering prevention, serially mediated by ascription of responsibility and personal norms, was negative (*β* = − 0.02, *p* = 0.02, 95% CI [− 0.04, − 0.003]), supporting hypothesis 5. Consistent with hypotheses 6 and 7, the indirect relationship between injunctive norms and personal norms via ascription of responsibility was positive (*β* = 0.05, *p* < 0.001, 95% CI [0.03, 0.08]), as was the serial indirect relationship between injunctive norms and littering prevention via ascription of responsibility and personal norms (*β* = 0.04, *p* = 0.001, 95% CI [0.02, 0.06]). Finally, the unstandardized indices of moderated mediation showed that, as injunctive norms strengthened, the indirect relationship between descriptive norms and personal norms via ascription of responsibility was less negative (index = 0.04, 95% CI [0.02, 0.05]). We observed the same moderation of the serial indirect relationship between descriptive norms and littering prevention via ascription of responsibility and personal norms (index = 0.03, 95% CI [0.01, 0.04]), supporting hypotheses 8 and 9 (see Appendix 6).

## Discussion and implications

This study examined the relationships between descriptive and injunctive norms and ascription of responsibility for littering prevention behavior. As we predicted, descriptive norms were negatively related and injunctive norms were positively related to ascription of responsibility. Our finding of a significant interaction effect suggests that strong injunctive norms may reduce the negative effect of descriptive norms on ascription of responsibility. These linkages form the core of the model of norm-regulated responsibility, which also provides new pathways linking social norms with personal norms and littering prevention behavior. We relate our findings to prior research and suggest avenues for future work.

First, we identified a new mechanism negatively linking descriptive norms with personal norms and littering prevention behavior. This may seem on its surface a simple replication of prior studies showing a boomerang effect of descriptive norms^73,74^. However, those studies mainly showed an effect of norm compliance. For instance, if a person is extremely environmentally friendly and everyone they meet is less environmentally friendly than they are, then descriptive normative influence would be away from being environmentally friendly^75,76^. In that special case, a descriptive normative message can result in less desirable behavior, opposite the intended effect. Although we found a negative linkage between descriptive norms and littering prevention behavior, it does not show the same kind of boomerang effect. We think the term “boomerang effect” still fits, but the mechanism has to do with the ascription of responsibility rather than norm compliance.

As we argued, the negative linkage between descriptive norms and littering prevention behavior is because descriptive norms may relieve individuals of the urgency to act, contributing to a denial of responsibility. This contrasts with prior research suggesting positive linkages among descriptive norms, personal norms, and littering prevention behavior due to the internalization of social norms and the function of descriptive norms in guiding accurate behaviors^15–17^. However, these arguments do not account for desirable behaviors like signing a petition or recycling where weak descriptive norms may sometimes encourage the behavior^49,50^. As we argued, the inaction of others could motivate individuals to pursue a collective goal that would otherwise not be achieved^49,50^. Alternatively, and consistent with our findings, descriptive norms could negatively influence behavior by diminishing ascription of responsibility. Specifically, strong descriptive norms may contribute to responsibility denial when individuals presume that many others are already engaged in the desirable behavior, thus reducing the need for any one individual to act.

Second, we found that injunctive norms were positively related to ascription of responsibility, personal norms, and littering prevention behavior, which is straightforward. These linkages might be due to the instructive nature of injunctive norms, the moral correctness injunctive norms may imply, and the social sanctions that may arise from norm-noncompliance^8^. These mechanisms enhance the necessity of individuals acting, leading to stronger feelings of responsibility. This is in contrast with the mechanism of descriptive norms, which have less to do with moral correctness about who *should* act and more to do with information about who *is* acting. Here we have returned to the descriptive normative effect because its contrast with the injunctive normative effect highlights the influence of incongruent social norms at the core of the model of norm-regulated responsibility.

More important, we found that the stronger the injunctive norms, the weaker the negative effect of descriptive norms on the ascription of responsibility. One reason for this may be that strong injunctive norms emphasize the moral responsibility of individuals to act, making it more difficult for them to shift the responsibility to others, as might otherwise occur when they perceive strong descriptive norms. In contrast, when individuals perceive strong descriptive norms and weak injunctive norms, they can shift the responsibility to others without violating social expectations. Thus, an additional key feature of the model of norm-regulated responsibility is that the negative effect of descriptive norms on ascription of responsibility is conditioned on the level of the injunctive norms. We wish to contrast this with Göckeritz, et al.^45^, who found that a positive effect of descriptive norms on proenvironmental behavior was stronger when there was a strong injunctive norm. In either case, injunctive norms have a beneficial role to play in moderating descriptive norms. When norms directly predict behavior, injunctive norms amplify the positive effect of descriptive norms. When they predict the ascription of responsibility, as we examined, injunctive norms attenuate the negative effect of descriptive norms.

While there are likely many behavioral contexts where individuals perceive strong descriptive and injunctive norms, is it realistic to predict effects of *strong* descriptive norms and *weak* injunctive norms? We believe it is realistic in contexts like littering prevention in Singapore, where government regulations, such as littering fines, may cause many people to perform the behavior. However, those regulations create extrinsic motivation and do not instill a sense of moral obligation to avoid littering. Thus, it is a possible situation in which most people engage in a proenvironmental behavior but feel indifferent about it. Consistent with that argument, when the behaviors reflected in strong descriptive norms are not attributed to intrinsic motivations, descriptive norms are less effective in promoting proenvironmental behavior^77^.

Given these incongruent and conditional effects of social norms on ascription of responsibility, the model of norm-regulated responsibility offers new insights into understanding normative influences on littering prevention behavior and proenvironmental behavior. It accounts for why strong descriptive norms may sometimes decrease behavioral motivation and provides a novel and more comprehensive explanation of normative influence on proenvironmental behavior. The distinct mechanisms of descriptive and injunctive norms in explaining ascription of responsibility draw on nuanced theorization, classification, and analysis of normative influence. Not only do the mechanisms suggest competing pathways leading to ascription of responsibility, but that injunctive norms may reorient individuals to descriptive norms.

Though, we need to emphasize that this model’s effectiveness likely depends on the behavioral context. It is possible the predicted effects arise only contexts where the responsible party is ambiguous or it is easy for individuals to deny responsibility, as may occur with littering prevention behavior and proenvironmental behaviors. Thus, the model of norm-regulated responsibility may be a poor choice in explaining behaviors such as diet and exercise, where individuals must take a more active role in resolving personal health needs^78^. In that context, we would not expect strong descriptive norms to have an inhibitory effect on behavior because other people achieving their health goals does not resolve a personal need. Moreover, there could be many other variables that could interfere with the relationships between social norms and ascription of responsibility. For instance, social norms may be one way that individuals reflect on their responsibility and form personal norms, personal norms can also moderate the effects of social norms on behavior. Among individuals with strong personal norms, their commitment to the behavior will tend to override social normative pressures. Establishing boundary conditions would be worthy of examination in future studies.

From a practical perspective, this research offers some insights for the use of normative messages in environmental communication and public policy. Communication interventions often follow “the higher, the better” rule as guidance for normative message design, given the generally positive association between social norms and behavior^15,20^. The model of norm-regulated responsibility implies a different rule. In some contexts, a communication intervention may be most successful if it can emphasize weak descriptive norms—or somehow that people are not doing enough—and strong injunctive norms. This would create the need for individual action and a framework of social sanctions for the failure to act. Although there is some evidence this combination of norms is effective for encouraging prosocial behaviors^50^, there is a need for case studies validating its use in practice. Nevertheless, according to our current findings, policymakers and environmental educators may consider emphasizing injunctive norms more, aiming to mitigate potential negative effects stemming from descriptive norms.

Finally, we wish to note some limitations of this study. First, the cross-sectional design prevents us from making causal inferences. Future studies should conduct experiments that manipulate the levels of descriptive and injunctive norms. Though, that approach would face its own challenge in creating believable levels of incongruent norms. Second, due to the use of secondary analysis, some measurements are not ideal. For example, we used single item to measure injunctive norms. Related, responses to the measures of descriptive and injunctive norms may have been influenced by the order in which the survey presented them. This is important to note because, when considering the combined effect of descriptive and injunctive norms, their mutual influence can distort their impact on the ascription of responsibility, personal norms, and behavioral intentions. Additionally, the wording of social norms measures can affect questionnaire responses^79^, a factor that we were unable to address in the current study. Third, this study focused on perceived descriptive norms and injunctive norms. Yet there are other types of social norms, such as collective norms and dynamic norms. Future studies may consider more types of social norms when investigating the role of social norms in ascription of responsibility. Moreover, norms are becoming increasingly abstract: people may share the notion of abstract values but their interpretation and manifestation of norms in concrete actions may vary^80^. In such situations, exploring how various descriptive norms and injunctive norms interact to shape people’s personal responsibility and actions becomes an interesting and important issue. Fourth, it is possible that the relationships found in this study depend on the national and cultural context. Future work using the model of norm-regulated responsibility should conduct cross-culture comparisons or, at least, account for individual-level cultural orientation. Finally, we recognize that social desirability bias may have affected our measures. Given the behavioral context, participants may have overreported their littering prevention. The use of hypothetical scenarios aimed to reduce this bias. Future research could go a step further with field experiments and unobtrusive observations of actual behavior.

## Conclusions

This study examined the relationships between descriptive and injunctive norms and ascription of responsibility for littering prevention behavior. As we predicted, descriptive norms were negatively related and injunctive norms were positively related to ascription of responsibility. Our finding of a significant interaction suggests that strong injunctive norms may reduce the negative effect of descriptive norms on ascription of responsibility. These linkages form the core of the model of norm-regulated responsibility, which also provides new pathways linking social norms with personal norms and littering prevention behavior. We contextualize our findings within the framework of existing research and current waste management practices, while also outlining potential directions for future investigations.

### Supplementary Information

Supplementary Information.

## Data Availability

The data that support the findings of this study are available from the corresponding author upon reasonable request.
