# Supplementary material for: The model of norm-regulated responsibility for proenvironmental behavior in the context of littering prevention
Source: Sci Rep. 2024 Apr 23;14:9289. doi: 10.1038/s41598-024-60047-0 (PMC11039738; doi:10.1038/s41598-024-60047-0)
Supplement: Supplementary file 1 — Supplementary Information. [file 41598_2024_60047_MOESM1_ESM.docx]

**Appendix 1**

*Literature review about the role of social norms and ascription of responsibility in the context of littering.*

| **Authors (Year)** | **Year** | **Title** | **Model** | **Area** | **Methodology** | **Statistical techniques** | **Variables** | **Measurements/manipulation of social norms or ascription of responsibility** | **Main findings on social norms or ascription of responsibility** |
| --- | --- | --- | --- | --- | --- | --- | --- | --- | --- |
| Sun, H., Yang, F., & Guo, W.^1^ | 2024 | Factors influencing hikers’ litter behavior in national park in China | The norm activation model | China | Survey | Regression and SEM | Awareness of consequences, ascription of responsibility, personal norms, environmental knowledge, connectedness to nature, pro-environmental behaviors, gender, age, education | Items of ascription of responsibility: - Every hiker is jointly responsible for litter in this national park  - Every hiker is partly responsible for litter in this national park  - Every hiker must take responsibility for litter problems in this national park | Ascription of responsibility was positively related to personal norms and behavior intention |
| Chaudhary, A. H., Polonsky, M. J., & McClaren, N.^2^ | 2023 | Social norms and littering – The role of personal responsibility and place attachment at a Pakistani beach | The focus theory of normative conduct | Pakistani | Survey | SEM and PROCESS model 7 | Injunctive norms, descriptive norms, place attachment, personal norms, antilittering behavioral intention | Items of injunctive norms - Most of my friends think I should perform behaviors that are safe for the environment - Most of my friends think I should not litter - Most of my neighbours think I should perform behaviors that are safe for the environment - Most of my neighbours think I should not litter - Most of my co-workers think I should perform behaviors that are safe for the environment - Most of my co-workers think I should not litter - Most of my family think I should perform behaviors that are safe for the environment - Most of my family think I should not litter - The leaders of my community encourage us all to be good to the environment  Items of descriptive norms - People around you try not to litter, although this might require more effort - People around you try to dispose litter properly, although this might require more time - People around you make an effort to avoid littering behavior while travelling - People around you get out of their comfort to avoid littering behavior | Descriptive and injunctive norms were positively related to personal norms. Place attachment strengthened the effect of descriptive norms. |
| Adjei, M., Song, H., Nketiah, E., Adu-Gyamfi, G., Obuobi, B., & Cifuentes-Faura, J. ^3^ | 2023 | Littering Prevention in Ghana: The Mediating and Moderating Effect of Awareness of Consequence with the Theory of Value-Belief-Norm | The theory of value-belief-norm | Ghana | Survey | SEM | Biospheric value, egoistic values, altruistic values, new environmental paradigm, awareness of consequence, ascribed of responsibility, personal norm, preventing littering behavior | - | Ascription of responsibility was positively related to personal norms |
| Chaudhary, A. H., Polonsky, M. J., & McClaren, N.^4^ | 2023 | Reducing Plastic Pollution Using Norms Perspective: Integration of Moral Position and Place Attachment | The focus theory of normative conduct | Pakistan | In-depth interview | - | Injunctive norms, descriptive norms, place attachment, idealism/relativism, personal norms, antilittering behavioral intentions | - | Social norms moderated the relationships between place attachment and littering behavior, and the relationship between ambiguous moral position and littering behavior. |
| Ojedokun, O., Henschel, N., Arant, R., & Boehnke, K.^5^ | 2022 | Applying the theory of planned behavior to littering prevention behavior in a developing country (Nigeria) | The theory of planned behavior | Nigeria | Survey | SEM | Descriptive norm, injunctive norm, cognitive attitude, emotional attitude, littering perceived behavioral control, littering prevention intention, littering prevention behavior | A sample item of descriptive norms: - Most people who are important to me engage in littering prevention actions in public places.  A sample item of injunctive norms: - If I do not take littering prevention actions, family members and friends who are important to me will disapprove. | The relationship between subjective norms and littering prevention intention was marginally significant |
| Zhang, A., Pang, B., Kim, J., Nguyen, T. M., & Nham, P. T. ^6^ | 2022 | An explorative study of psychological and social factors impacting littering behavior in Vietnam | The theory of planned behavior | Vietnam | Survey | SEM | Injunctive norms, descriptive norms, environmental consciousness, perceived behavioral control, connectedness to nature, attitude towards littering, intention to stop littering | Items of injunctive norms: - If I litter in public place, people who are important to me would - Most people who are important to me think that littering in public place is. - Most people who are important to me think that I should littering in public place. Items of descriptive norms: - Most members of my community currently stop littering in public place. - How much agreement is there amongst members of your community that stop littering in public place is a good thing? | The descriptive norms moderated the relationship between connectedness to nature and intention to stop littering, and the relationship between attitude and intention to stop littering. Injunctive norms moderated the relationship between perceived behavioral control and the intention to stop littering. |
| Gangl, K., Walter, A., & Van Lange, P. A. M. ^7^ | 2022 | Implicit reminders of reputation and nature reduce littering more than explicit information on injunctive norms and monetary costs | - | Vienna | Field experiment | ANOVA | Monetary information, depiction of the injunctive norm, watching eyes, nature picture;  Control variables: waste in front of the waste disposal area, residual waste is full, paper waste is full, residents per waste area, available waste capacity per resident, poster disappeared/is destroyed, cigarettes on the floor, bulky waste. | The manipulation of injunctive norms: Visual explanation of the injunctive norm and the negative consequences. | The norm condition was not significantly different from the control condition. |
| Rosenthal, S., & Max, S. C.^8^ | 2022 | Anticipated guilt and anti-littering civic engagement in an extended norm activation model | The norm activation model | Singapore | Survey | SEM | Awareness of consequences, community attachment, ascription of responsibility, personal norm, anticipated shame/embarrassment, anticipated guilt, self-managing behavior, other-managing behavior, civic engagement. | Items of ascription of responsibility: - It is up to individuals to keep the environment clean. - Individuals are responsible for dealing with litter. | Ascription of responsibility was positively related to personal norms |
| Fenitra, R. M., Premananto, G. C., Sedera, R. M. H., Abbas, A., & Laila, N. ^9^ | 2022 | Environmentally responsible behavior and Knowledge-Belief-Norm in the tourism context: The moderating role of types of destinations | The knowledge-belief-norm model | Indonesia | Survey | SEM | biospheric value, environmental knowledge, new environmental paradigm, awareness of the consequences, ascription of responsibility, personal norm, environmentally responsible behavior intention | Items of ascription of responsibility: - I am responsible for the impacts of litter on the environment as a tourist.  - I am responsible for the impacts of litter on the environment. - I am responsible for minimizing the impacts of litter on the environment as a tourist. | Ascription of responsibility was positively related to personal norms |
| Bergquist, M., Blumenschein, P., Karinti, P., Köhler, J., Ramos, É. M. S., Rödström, J., & Ejelöv, E. ^10^ | 2021 | Replicating the focus theory of normative conduct as tested by Cialdini et al. (1990) | The focus theory of normative conduct | The United States | Experiment | ANOVA | Study 1: descriptive norms, littering  Study 2: descriptive norms, littering  Study 3: descriptive norms, environmental setting, littering | Manipulations of descriptive norms: Study 1: the amount of litter in the environment Study 2: the number of pieces of litter in the environment Study 3: the amount of litter in the environment | In clean environments, there was less littering compared to littered ones. Adding just one piece of litter to a clean environment increases littering. The presence of an anti-littering norm and the belief that littering is uncommon was more noticeable when there's only one piece of litter compared to a completely clean environment. However, as litter accumulated, the influence of the anti-littering norm seems to weaken. |
| Farage, L., Uhl-Haedicke, I., & Hansen, N. ^11^ | 2021 | Problem awareness does not predict littering: A field study on littering in the Gambia | The norm activation model | Gambia | Survey | SEM | Awareness of consequences, ascription of responsibility, personal norm, anti-littering behavioral intention, littering behavior | - | Ascription of responsibility was positively related to personal norms |
| Esfandiar, K., Dowling, R., Pearce, J., & Goh, E. ^12^ | 2021 | What a load of rubbish! The efficacy of theory of planned behavior and norm activation model in predicting visitors' binning behavior in national parks | The theory of planned behavior, the norm activation model | Western Australia | Survey | SEM | Social norms, attitudes, awareness of consequences, personal norms, perceived behavioral control, binning behavior | Items of social norms: - Most important people in my life (if they were in this park) would encourage me to bin my litter. - Most important people in my life (if they were in this park) would disapprove if I littered in YNP. - Those with whom I visit YNP would care about my binning behavior. | Social norms were positively related to personal norms and behavior |
| Park, H. S., Ulusoy, E., Choi, S. Y., & Lee, H. E. ^13^ | 2020 | Temporal Distance and Descriptive Norms on Environmental Behaviors: A Cross-Cultural Examination of Construal-Level Theory | The construal-level theory | The United States and Korea | Survey | ANOVA and correlation | Perceived descriptive norms; situational descriptive norms and behavioral intentions; littering/recycling behavior, temporal distance | Items of perceived descriptive norms:  - Many people in the U.S. (Korea) carelessly discard litter. - Many U.S. (Korean) citizens are willing to litter. - Littering is a common behavior that people in the U.S. (Korea) engage in. - Many U.S. (Korean) citizens throw trash on the street.  Manipulation of situational descriptive norms:  A passage letting the readers imaging a place with trash | The perceived descriptive norms concerning recycling and littering was not associated with intentions regarding recycling and littering. However, when the data from both the United States and Korea were aggregated under conditions with no specified time indication, perceived descriptive norms was negatively related to intentions to litter. |
| Rosenthal, S., & Ho, K. L. ^14^ | 2020 | Minding other people's business: Community attachment and anticipated negative emotion in an extended norm activation model | The norm activation model | Singapore | Survey | SEM | Awareness of consequences, ascription of responsibility, personal norm, community attachment, anticipated negative emotion, self-managing behavior, other-managing behavior | Items of ascription of responsibility: - It is up to individuals to keep the environment clean. - Individuals are responsible for dealing with litter. | Ascription of responsibility was positively related to personal norms |
| Esfandiar, K., Dowling, R., Pearce, J., & Goh, E. ^15^ | 2020 | Personal norms and the adoption of pro-environmental binning behavior in national parks: An integrated structural model approach | The theory of planned behavior, the norm activation model | Iran | Survey | SEM | Social norms, attitudes, awareness of consequences, personal norms, perceived behavioral control, binning behavior | Items of social norms: - Most important people in my life (if they were in this park) would encourage me to bin my litter.  - Most important people in my life (if they were in this park) would encourage me to bin my litter.  - Those whom I go to SNP would care about my binning behavior. | Social norms were positively related to personal norms and behavior |
| Esfandiar, K., Pearce, J., & Dowling, R.^16^ | 2019 | Explaining littering prevention among park visitors using the Theory of Planned Behavior and Norm Activation Model | The theory of planned behavior, the norm activation model | Indonesia | Survey | SEM | Awareness of consequences; ascription of responsibility; personal norm; attitude toward behavior; subjective norm; perceived behavior control; environmentally responsible behavior intention | Items of subjective norm: - Most people who are important to me engage in littering prevention actions in national parks. - If I do not take littering prevention actions, family members and friends who are important to me will disapprove.  Items of ascription of responsibility: - I am responsible for the impacts of litter on the environment. - I am responsible for minimizing the impacts of litter on the environment. I am responsible for minimizing the impacts of litter on the environment | Ascription of responsibility was positively related to personal norms. Social norms were not related to environmentally responsible behavior intention |
| Esfandiar, K., Pearce, J., & Dowling, R. ^17^ | 2019 | Personal norms and pro-environmental binning behavior of visitors in national parks: The development of a conceptual framework | The norm activation model | - | - | - | Social norms, attitudes, awareness of consequences, pro-environmental personal norms, perceived behavioral control, pro-environmental binning intention, binning behavior | - | - |
| Cialdini, R. B. ^18^ | 2003 | Crafting Normative Messages to Protect the Environment | - | - | - | - | - | - | To fully harness the effectiveness of normative appeals, it is necessary to synchronize descriptive norms with injunctive norms. |
| Kallgren, C. A., Reno, R. R., & Cialdini, R. B. ^19^ | 2000 | A Focus Theory of Normative Conduct: When Norms Do and Do not Affect Behavior | The focus theory of normative conduct | The United States | Experiment | ANOVA | Study 1: Arousal, related to antilittering norm, percent littering Study 2: Degree of norm focus, number of handbills, percent littering | Manipulations of descriptive norms: Study 1: Exemplars in a diary passage; Study 2: A confederate picked up a crumpled fast-food bag. | Study 1: Norms have a potent impact on behavior only to the extent that the norms relevant to the behavior in question are focal or salient.  Study 2: Participants whose attention was focused on the anti-littering norm became less willing to violate the norm. |
| Cialdini, R. B., Reno, R. R., & Kallgren, C. A. ^20^ | 1990 | A focus theory of normative conduct: Recycling the concept of norms to reduce littering in public places. | The focus theory of normative conduct | The United States | Experiment | ANOVA | Study 1: Norm salience, direction of descriptive norm, littering Study 2: the number of pieces of litter in the environment, littering Study 3: the amount of litter in the environment, littering Study 4: the configuration of litter in the environment, littering Study 5: proximity to the injunctive norm against littering, littering | Manipulations of descriptive norms: Study 1: the amount of litter in the environment, littering Study 2: the number of pieces of litter in the environment, littering Study 3: the amount of litter in the environment, littering Study 4: the amount of litter in the environment, Injunctive norm: Upswept Litter/(Swept Litter) Manipulations of injunctive norms: Study 5: Normative message describing what people should do | Focusing subjects on either the descriptive norms or the injunctive norms regarding littering caused their littering decisions to change only in accordance with the dictates of the more salient type of norm at that time. |
| Note. SEM = Structural Equation Modeling | | | | | | | | | |

**References**

1 Sun, H., Yang, F. & Guo, W. Factors influencing hikers’ litter behavior in national park in China. *Front. For. Global Change* **6** (2024).

2 Chaudhary, A. H., Polonsky, M. J. & McClaren, N. Social norms and littering – The role of personal responsibility and place attachment at a Pakistani beach. *Global Environ. Change* **82**, 102725, doi:<https://doi.org/10.1016/j.gloenvcha.2023.102725> (2023).

3 Adjei, M. *et al.* Littering Prevention in Ghana: The Mediating and Moderating Effect of Awareness of Consequence with the Theory of Value-Belief-Norm. *Soc. Nat. Resour.*, 1-24, doi:10.1080/08941920.2023.2291782.

4 Chaudhary, A. H., Polonsky, M. J. & McClaren, N. in *Socially Responsible Plastic* Vol. 19 *Developments in Corporate Governance and Responsibility* (eds David Crowther & Farzana Quoquab) 123-149 (Emerald Publishing Limited, 2023).

5 Ojedokun, O., Henschel, N., Arant, R. & Boehnke, K. Applying the theory of planned behaviour to littering prevention behaviour in a developing country (Nigeria). *Waste Manage. (Oxford)* **142**, 19-28, doi:<https://doi.org/10.1016/j.wasman.2022.02.006> (2022).

6 Zhang, A., Pang, B., Kim, J., Nguyen, T.-M. & Nham, P. T. An explorative study of psychological and social factors impacting littering behavior in Vietnam. *Front. Psychol.* **13** (2022).

7 Gangl, K., Walter, A. & Van Lange, P. A. M. Implicit reminders of reputation and nature reduce littering more than explicit information on injunctive norms and monetary costs. *J. Environ. Psychol.* **84**, 101914, doi:<https://doi.org/10.1016/j.jenvp.2022.101914> (2022).

8 Rosenthal, S. & Yu, M. S. C. Anticipated guilt and anti-littering civic engagement in an extended norm activation model. *J. Environ. Psychol.* **80**, doi:10.1016/j.jenvp.2022.101757 (2022).

9 Fenitra, R. M., Premananto, G. C., Sedera, R. M. H., Abbas, A. & Laila, N. Environmentally responsible behavior and Knowledge-Belief-Norm in the tourism context: The moderating role of types of destinations. *International Journal of Geoheritage and Parks* **10**, 273-288, doi:<https://doi.org/10.1016/j.ijgeop.2022.05.001> (2022).

10 Bergquist, M. *et al.* Replicating the focus theory of normative conduct as tested by Cialdini et al. (1990). *J. Environ. Psychol.* **74**, doi:10.1016/j.jenvp.2021.101573 (2021).

11 Farage, L., Uhl-Haedicke, I. & Hansen, N. Problem awareness does not predict littering: A field study on littering in the Gambia. *J. Environ. Psychol.* **77**, 101686, doi:<https://doi.org/10.1016/j.jenvp.2021.101686> (2021).

12 Esfandiar, K., Dowling, R., Pearce, J. & Goh, E. What a load of rubbish! The efficacy of theory of planned behaviour and norm activation model in predicting visitors’ binning behaviour in national parks. *J. Hosp. Tour. Manag.* **46**, 304-315, doi:<https://doi.org/10.1016/j.jhtm.2021.01.001> (2021).

13 Park, H. S., Ulusoy, E., Choi, S. Y. & Lee, H. E. Temporal Distance and Descriptive Norms on Environmental Behaviors: A Cross-Cultural Examination of Construal-Level Theory. *SAGE Open* **10**, 2158244020914576, doi:10.1177/2158244020914576 (2020).

14 Rosenthal, S. & Ho, K. L. Minding other people's business: Community attachment and anticipated negative emotion in an extended norm activation model. *J. Environ. Psychol.* **69**, doi:10.1016/j.jenvp.2020.101439 (2020).

15 Esfandiar, K., Dowling, R., Pearce, J. & Goh, E. Personal norms and the adoption of pro-environmental binning behaviour in national parks: an integrated structural model approach. *Journal of Sustainable Tourism* **28**, 10-32, doi:10.1080/09669582.2019.1663203 (2020).

16 Fenitra, R. M., Laila, N., Premananto, G. C., Abbas, A. & Sedera, R. M. H. Explaining littering prevention among park visitors using the Theory of Planned Behavior and Norm Activation Model. *International Journal of Geoheritage and Parks* **11**, 39-53, doi:<https://doi.org/10.1016/j.ijgeop.2022.11.002> (2023).

17 Esfandiar, K., Pearce, J. & Dowling, R. Personal norms and pro-environmental binning behaviour of visitors in national parks: the development of a conceptual framework. *Tourism Recreation Research* **44**, 163-177, doi:10.1080/02508281.2019.1580936 (2019).

18 Cialdini, R. B. Crafting Normative Messages to Protect the Environment. *Current Directions in Psychological Science* **12**, 105-109, doi:10.1111/1467-8721.01242 (2003).

19 Kallgren, C. A., Reno, R. R. & Cialdini, R. B. A Focus Theory of Normative Conduct: When Norms Do and Do not Affect Behavior. *Personality and Social Psychology Bulletin* **26**, 1002-1012, doi:10.1177/01461672002610009 (2000).

20 Cialdini, R. B., Reno, R. R. & Kallgren, C. A. A focus theory of normative conduct: Recycling the concept of norms to reduce littering in public places. *J. Pers. Soc. Psychol.*, 1015-1026, doi:10.1037/0022-3514.58.6.1015 (1990).

**Appendix 2**

*Measurement, factor loadings, and reliability estimates of model variables.*

| Construct/Item | Wording | M | SD | λ |
| --- | --- | --- | --- | --- |
| **Descriptive Norms (AVE = .54, CR = .69, VIF = 1.42)** | |  |  |  |
| DN1 | Most people in my community avoid littering. | 3.46 | .87 | .89 |
| DN2 | I rarely see people littering in my neighborhood. | 3.19 | 1.04 | .55 |
| **Injunctive Norms (VIF = 1.44)** | |  |  |  |
| IN1 | People in my community disapprove of littering. | 3.61 | .77 |  |
| **Ascriptive of Responsibility (AVE = .62, CR = .76, VIF = 1.34)** | | | | |
| AR1 | It is up to individuals to keep the environment clean. | 4.24 | .83 | .70 |
| AR2 | Individuals are responsible for dealing with litter. | 4.22 | .77 | .87 |
| **Personal Norms (AVE = .55, CR = .71, VIF = 2.15)** | | | | |
| PN1 | I have a moral obligation to avoid littering. | 4.23 | .72 | .75 |
| PN2 | I feel a personal responsibility not to litter. | 4.38 | .70 | .73 |
| **Litter Prevention (AVE = .50, CR = .75)** | |  |  |  |
| LP1 | You are returning your tray at a hawker centre and your plastic spoon falls on the ground next to the tray return. Do you pick up it up? | 4.49 | .84 | .68 |
| LP2 | You are discarding a wadded-up receipt and accidentally miss the bin. It lands on the ground, where there are other small pieces of rubbish. Do you pick up the receipt? | 4.27 | .95 | .67 |
| LP3 | You are exiting a stairwell and accidentally drop a small piece of paper that you were going to throw away. Nobody saw you drop it. Do you pick it up? | 4.36 | .89 | .77 |
| **Awareness of Consequences (AVE = .55, CR = .88, VIF = 1.04)** | | | | |
| AC1 | Litter has a negative effect on tourism. | 4.30 | .83 | .68 |
| AC2 | Litter results in wasted tax dollars. | 4.15 | .83 | .65 |
| AC3 | Litter is harmful to native species of wildlife. | 4.34 | .76 | .71 |
| AC4 | Litter pollutes the water supply. | 4.38 | .74 | .76 |
| AC5 | Litter creates unsanitary conditions. | 4.47 | .67 | .83 |
| AC6 | Litter attracts vermin, such as rats and cockroaches. | 4.55 | .67 | .82 |

*Note.* *M* = mean. *SD* = standard deviation of measurement items. λ = standardized factor loading from the measurement model. AVE = average variance extracted. CR = composite reliability. The measure of injunctive norm used a single item, so we report only the mean and standard deviation.

**Appendix 3**

*Correlation, variance, and covariance matrix.*

|  | 1 | 2 | 3 | 4 | 5 | 6 |
| --- | --- | --- | --- | --- | --- | --- |
| 1. Descriptive norms | **0.60** | 0.32 | 0.01 | 0.08 | 0.03 | 0.04 |
| 2. Injunctive norms | .54^***^ | **0.59** | 0.07 | 0.08 | 0.03 | 0.05 |
| 3. Ascription of responsibility | .03 | .15^***^ | **0.34** | 0.18 | 0.14 | 0.15 |
| 4. Personal norms | .18^***^ | .19^***^ | .57^***^ | **0.29** | 0.20 | 0.19 |
| 5. Litter prevention | .06 | .08^*^ | .41^***^ | .65^***^ | **0.32** | 0.14 |
| 6. Awareness of consequences | .09^**^ | .12^***^ | .47^***^ | .63^***^ | .44^***^ | **0.32** |

*Note.* The diagonal contains item variances. Covariances are above the diagonal. Correlations are below the diagonal. **p* < .05, ***p* < .01, ****p* < .001

**Appendix 4**

*Standardized path coefficients.*

| Path | *β* | *p* | 95% CI | |
| --- | --- | --- | --- | --- |
|  |  |  | Lower bound | Upper bound |
| DN 🡪 AR | -.09 | .02 | -.16 | -.01 |
| IN 🡪 AR | .14 | < .001 | .08 | .21 |
| AC 🡪 AR | .38 | < .001 | .39 | .53 |
| DN 🡪 PN | .12 | .01 | .03 | .21 |
| IN 🡪 PN | .02 | .48 | -.05 | .10 |
| AR 🡪 PN | .37 | < .001 | .28 | .45 |
| AC 🡪 PN | .45 | < .001 | .36 | .53 |
| DN 🡪 LP | -.05 | .24 | -.14 | .03 |
| IN 🡪 LP | -.03 | .50 | -.10 | .05 |
| PN 🡪 LP | .68 | < .001 | .63 | .74 |

*Note.* DN = descriptive norms. IN = injunctive norms. AR = ascription of responsibility. PN = personal norms. LP = litter prevention. AC = awareness of consequences. 95% CI is based on 1,000 bootstrap samples.

**Appendix 5**

*Main effects model and interaction effect model predicting ascription of responsibility.*

| Model | Path Estimate | |  | Log Likelihood Ratio Test | | | |
| --- | --- | --- | --- | --- | --- | --- | --- |
|  | *β* | *p* |  | Log Likelihood Ratio | *D* | *df* | *p* |
| Main effects |  |  |  | -22591.96 |  |  |  |
| Descriptive norms | -.09 | .02 |  |  |  |  |  |
| Injunctive norms | .14 | <.001 |  |  |  |  |  |
| Interaction effect |  |  |  | -22593.14 | 2.36 | 1 | 0.12 |
| Descriptive norms | -.57 | <.001 |  |  |  |  |  |
| Injunctive norms | .16 | <.001 |  |  |  |  |  |
| Descriptive norms  × injunctive norms | .10 | <.001 |  |  |  |  |  |

*Note*. *β* = standardized path estimate. *p* = *p*-value. *D* = log likelihood ratio. *df* = degrees of freedom of the log likelihood ratio.

**Appendix 6**

*Direct, indirect, and total effects.*

| Path | *β* | *p* | 95% CI | |
| --- | --- | --- | --- | --- |
|  |  |  | Lower bound | Upper bound |
| DN 🡪 AR 🡪 PN | -.03 | .02 | -.06 | -.004 |
| DN 🡪 PN (direct effect) | .12 | .01 | .03 | .20 |
| DN 🡪 PN (total effect) | .09 | .06 | -.002 | .18 |
| DN 🡪 AR 🡪 PN 🡪 LP | -.02 | .02 | -.04 | -.003 |
| DN 🡪 PN 🡪 LP | .08 | .01 | .02 | .14 |
| DN 🡪 LP (direct effect) | -.05 | .24 | -.14 | .03 |
| DN 🡪 LP (total indirect effect) | .06 | .06 | .000 | .12 |
| DN 🡪 LP (total effect) | .01 | .83 | -.07 | .09 |
| IN 🡪 AR 🡪 PN | .05 | < .001 | .03 | .08 |
| IN 🡪 PN (direct effect) | .02 | .54 | -.05 | .10 |
| IN 🡪 PN (total effect) | .08 | .06 | -.004 | .15 |
| IN 🡪 AR 🡪 PN 🡪 LP | .04 | .001 | .02 | .06 |
| IN 🡪 PN 🡪 LP | .02 | .54 | -.04 | .07 |
| IN 🡪 LP (direct effect) | -.03 | .47 | -.10 | .05 |
| IN 🡪 LP (total indirect effect) | .05 | .06 | -.002 | .11 |
| IN 🡪 LP (total effect) | .02 | .51 | -.04 | .11 |
| *Note.* DN = descriptive norms. IN = injunctive norms. AR = ascription of responsibility. PN = personal norms. LP = litter prevention. AC = awareness of consequences. 95% CI is based on 1,000 bootstrap samples. | | | | |
